# The Elements Pathology

**Published:** 1884-11

**Authors:** 


**The Elements of Pathology**. By EDWARD RINDFLEISCH, M. D., Professor of Pathological Anatomy in the University of Wursburg. Translated from the first German edition by WILLIAM H. MERCUR, M. D., University of Pennsylvania. Revised by JAMES TYSON, M. D., Professor of General Pathology and Morbid Anatomy in the University of Pennsylvania. Philadelphia: P. Blakiston, Son & Co., 1012 Walnut Street. 1884. Price, $2.00.

This work does not aim to be a text-book. Its purpose is simply to establish the natural groundwork which exists in this as well as in every natural science, and to place it in as clear a light as possible. Prof. Tyson's connection with the work is a guarantee that the American edition will fill a niche in the wants of the student and of others who may desire to become familiar with general pathological processes, viewed from the most modern standpoint. The importance of the study of pathology in the medical curriculum is now fully recognized, and we think this work facilitates its study.

